# Supplementary figures and images for: Extended regimen of a levonorgestrel/ethinyl estradiol transdermal delivery system: Predicted serum hormone levels using a population pharmacokinetic model
Source: PLoS One. 2022 Dec 27;17(12):e0279640. doi: 10.1371/journal.pone.0279640 (PMC9794042; doi:10.1371/journal.pone.0279640)

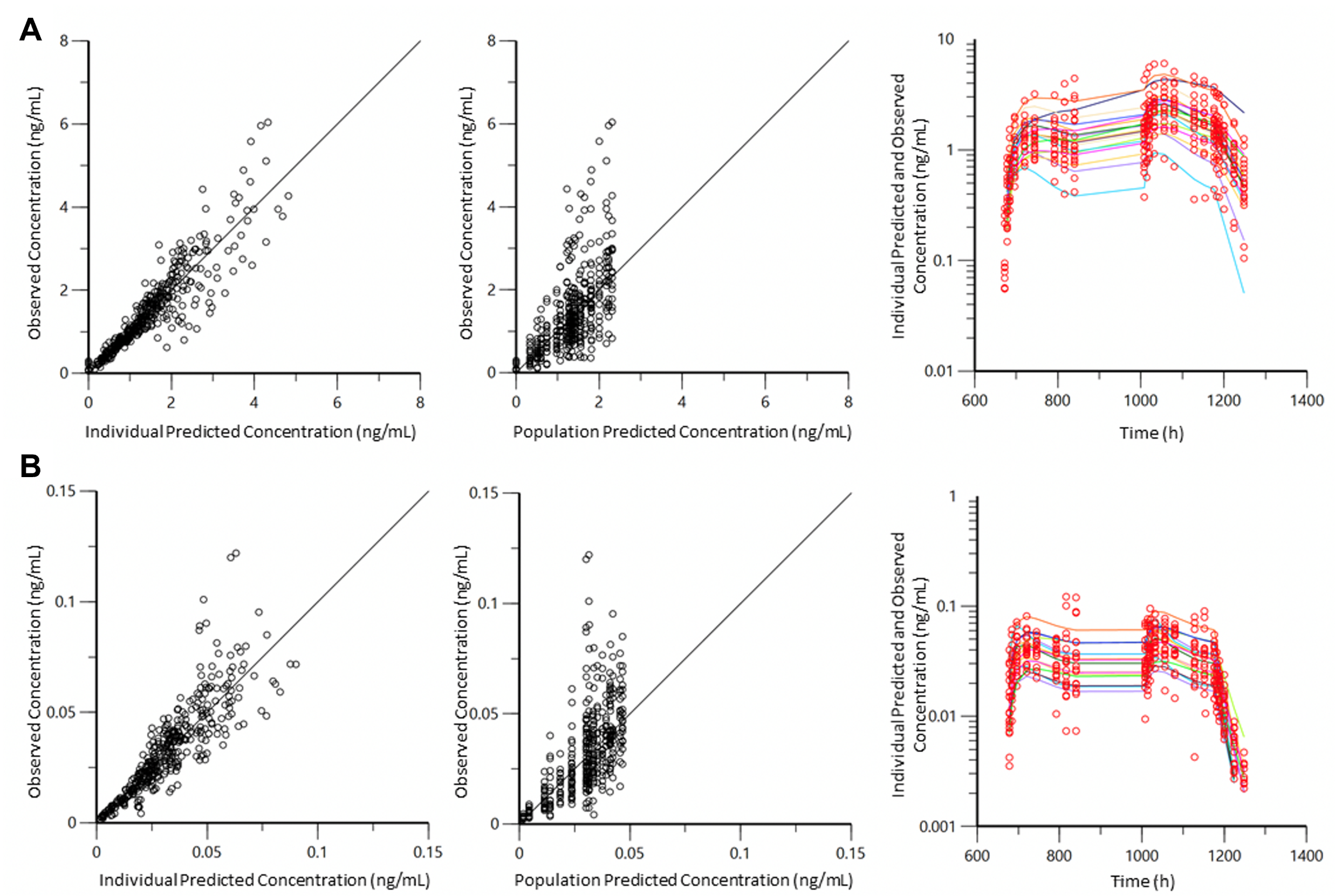

Supplement: S1 Fig — A. EE popPK model goodness-of-fit plots. B. LNG popPK model goodness-of-fit plots. EE, ethinyl estradiol; LNG, levonorgestrel; popPK, population pharmacokinetics. (TIF) [file pone.0279640.s001.tif]

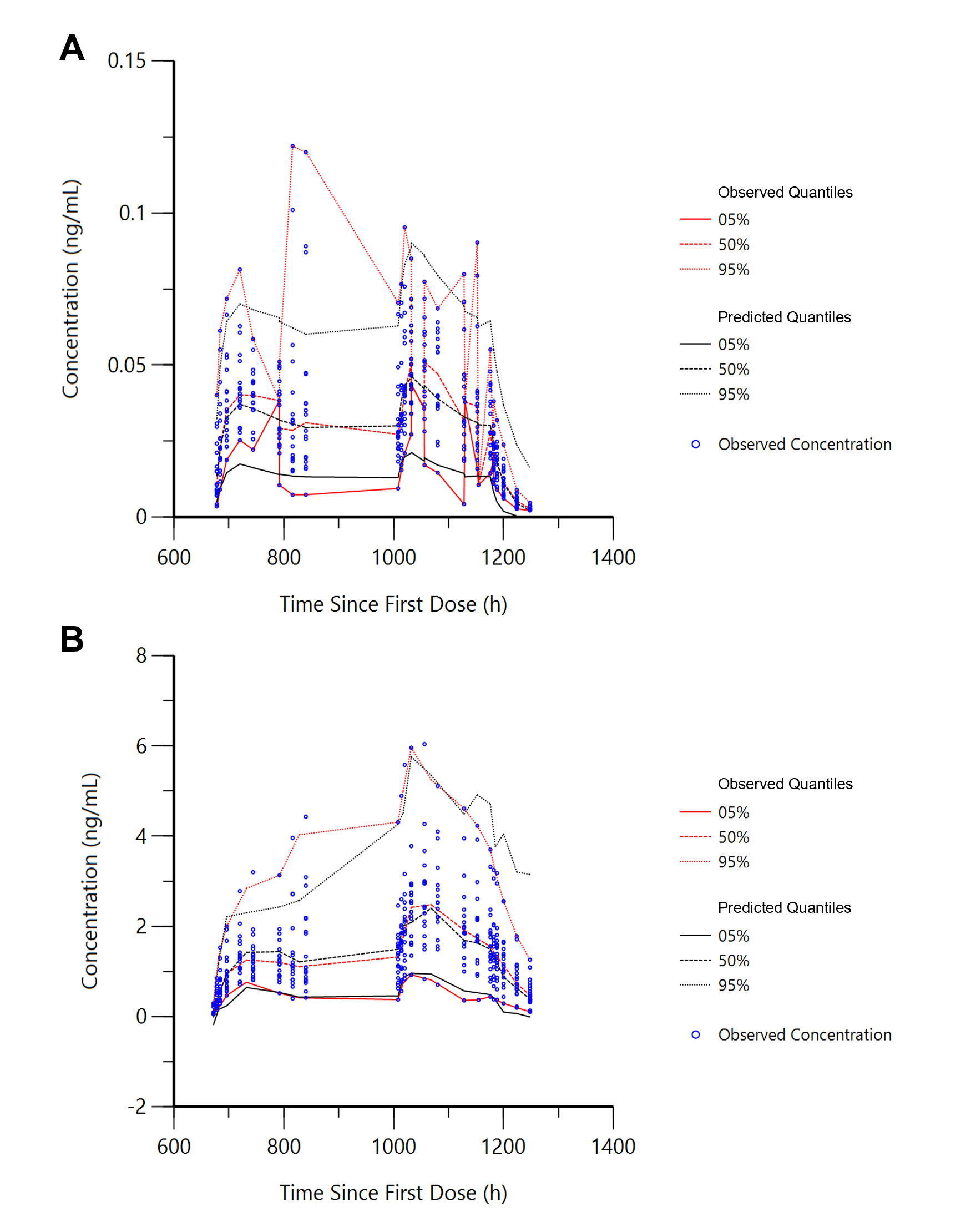

Supplement: S2 Fig — A. Visual Predictive Check for EE. B. Visual Predictive Check for LNG. EE, ethinyl estradiol; LNG, levonorgestrel. (TIF) [file pone.0279640.s002.tif]

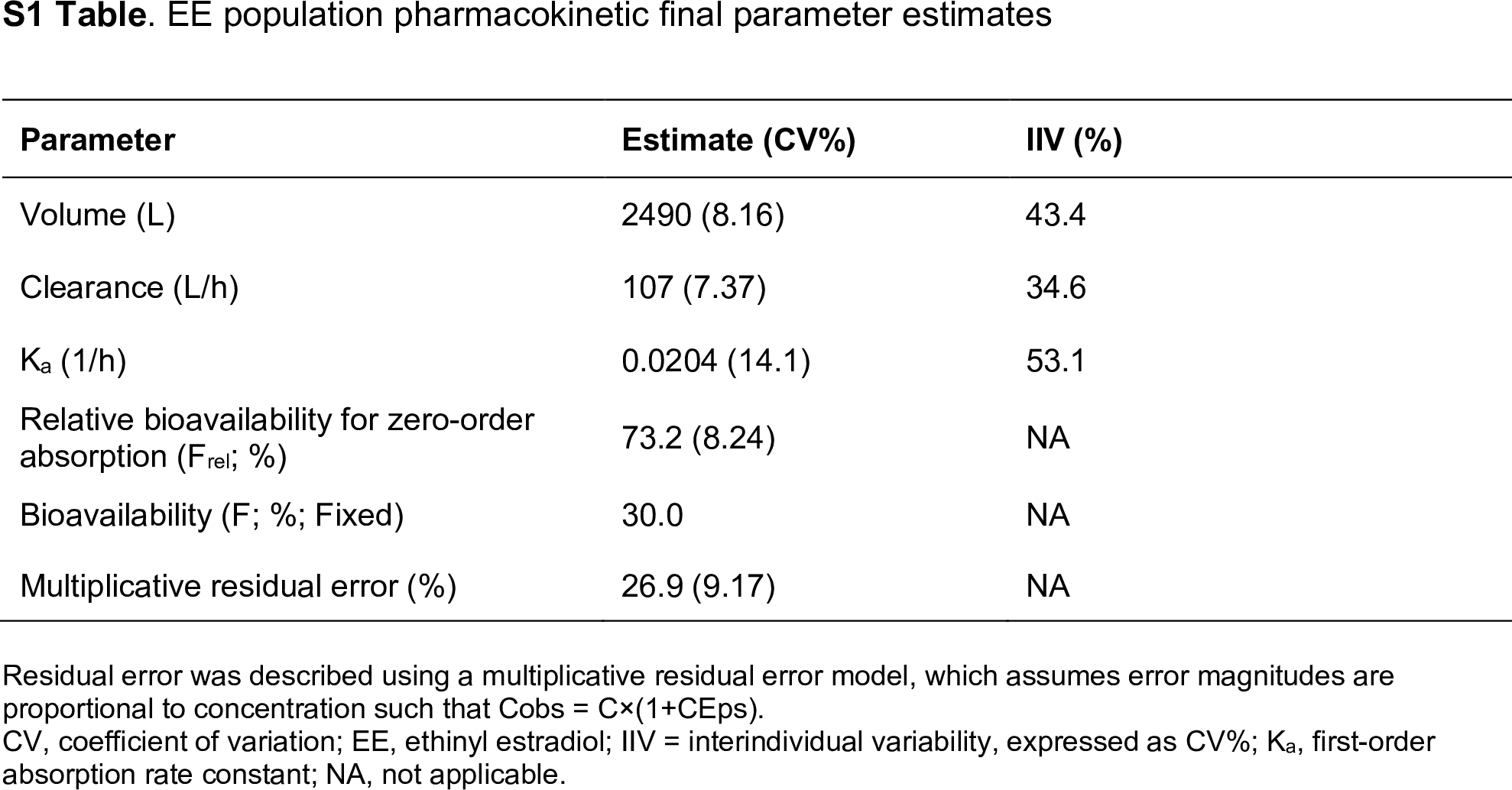

Supplement: S1 Table — (TIF) [file pone.0279640.s003.tif]

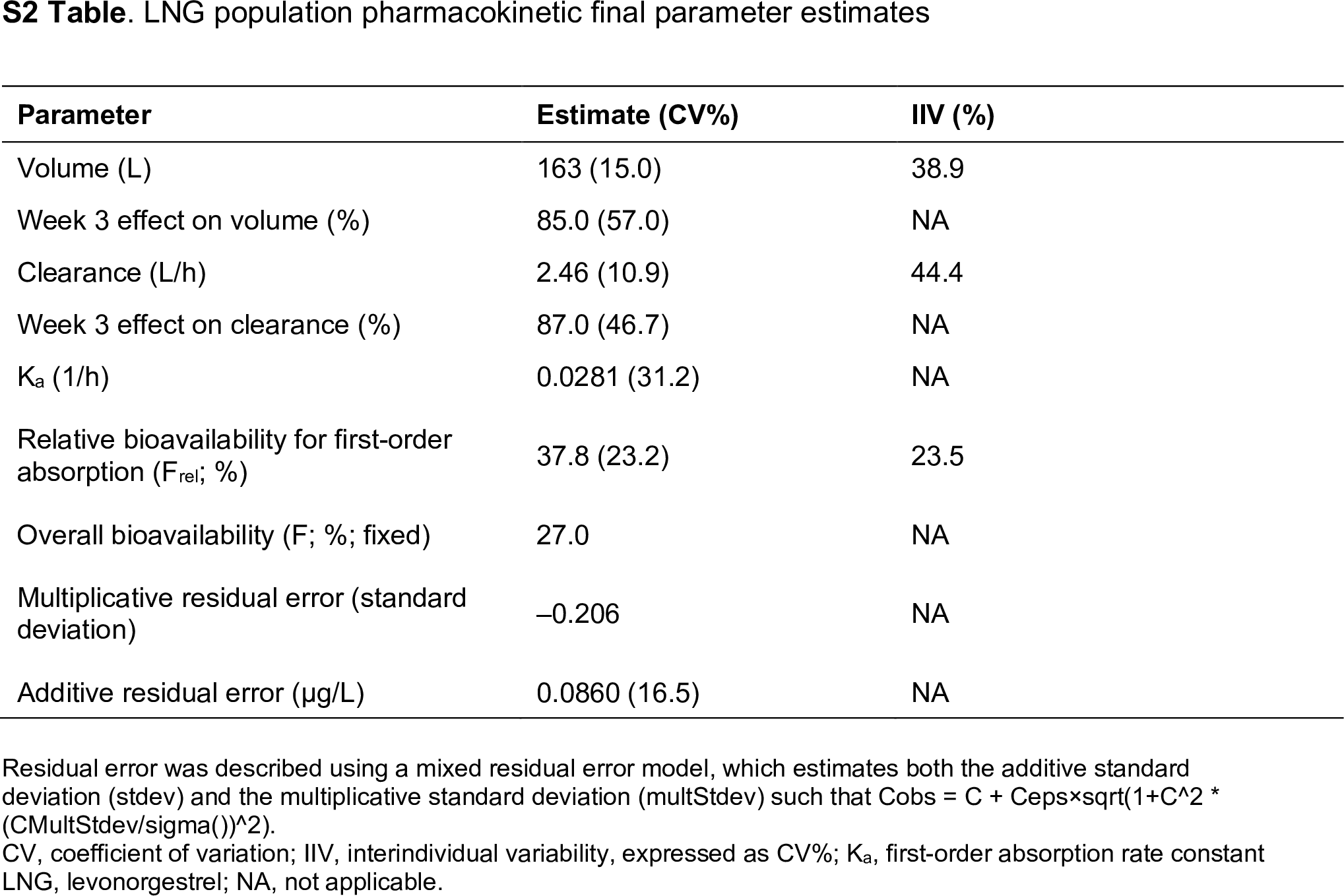

Supplement: S2 Table — (TIF) [file pone.0279640.s004.tif]

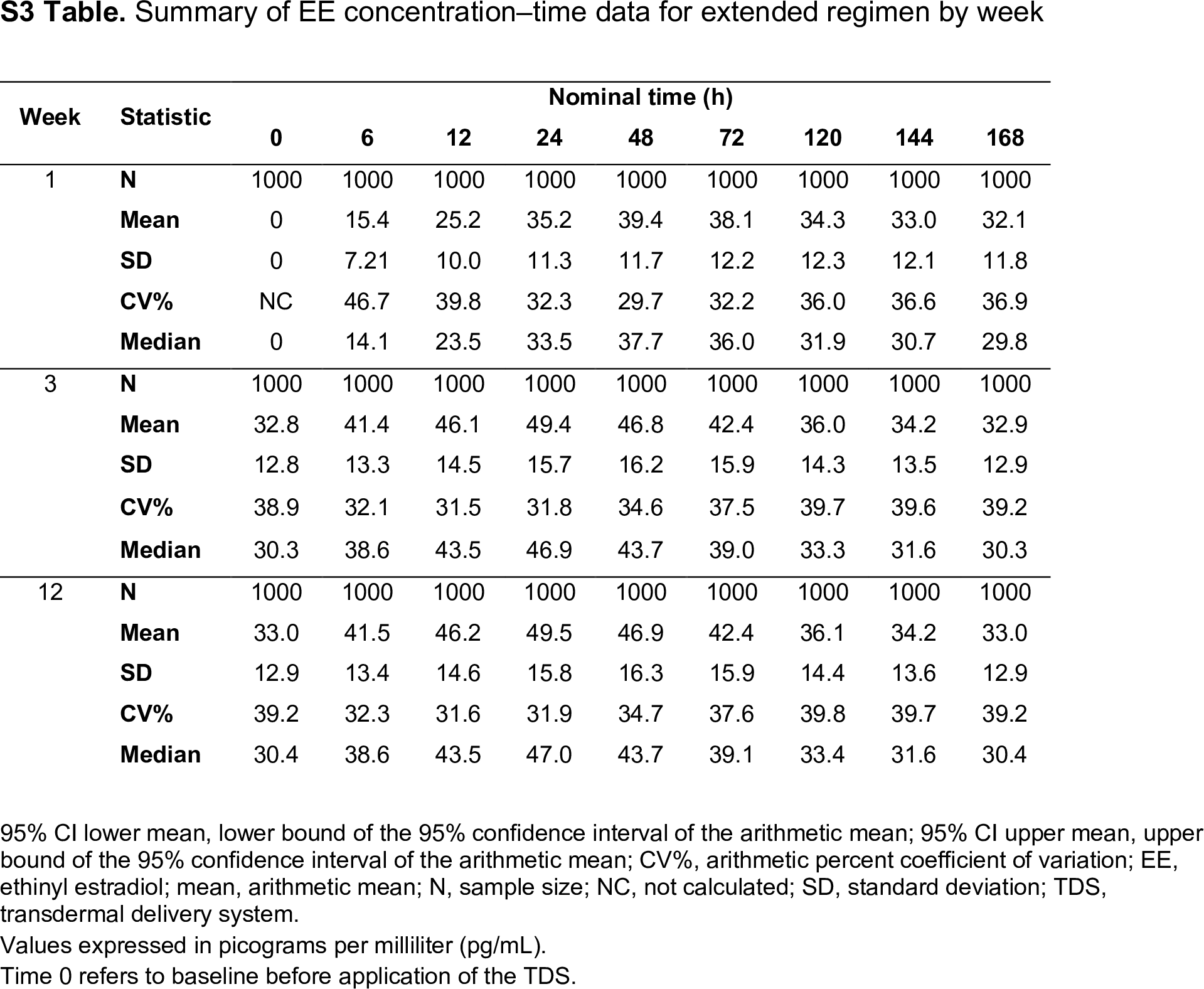

Supplement: S3 Table — (TIF) [file pone.0279640.s005.tif]

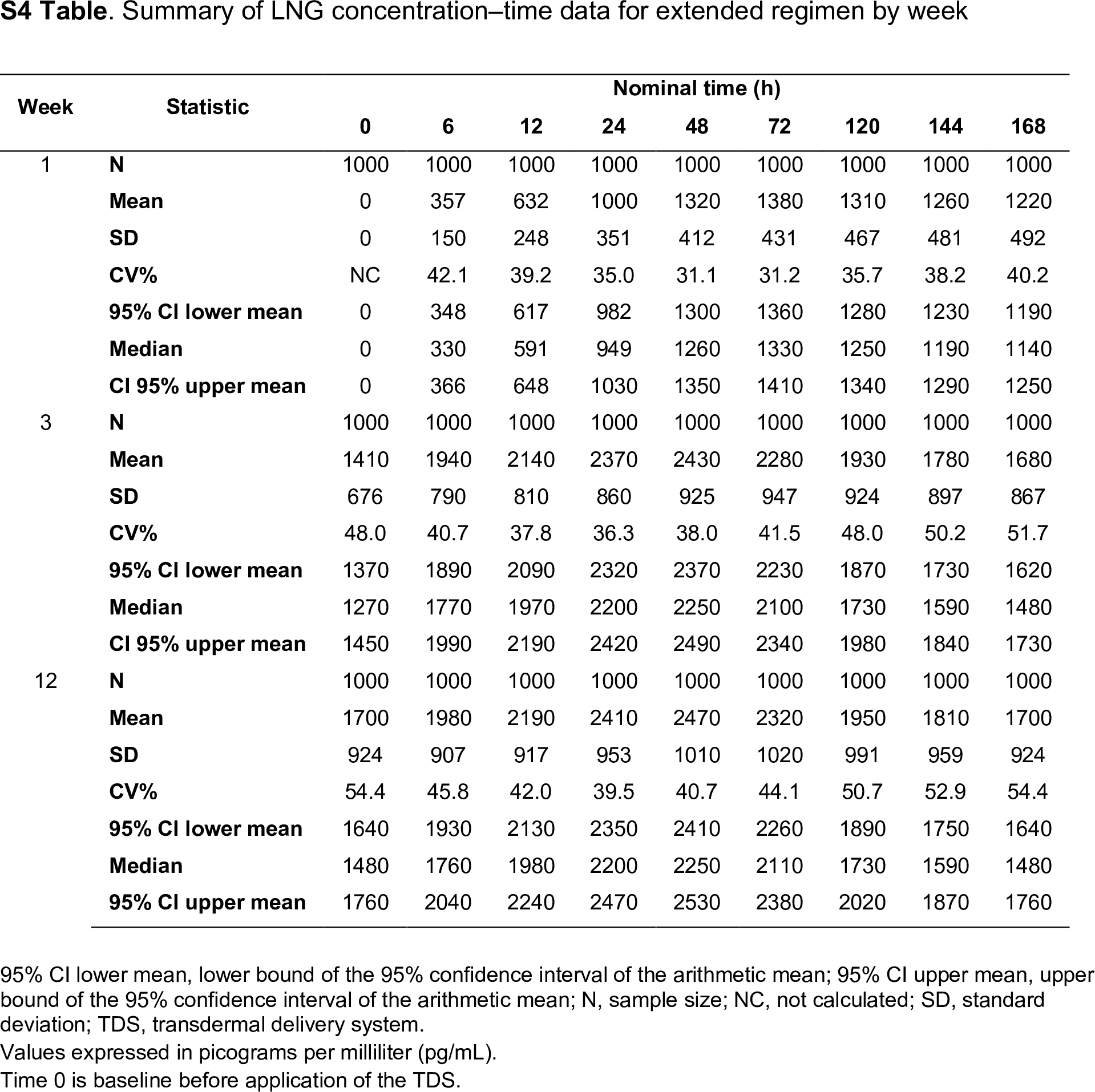

Supplement: S4 Table — (TIF) [file pone.0279640.s006.tif]
